# Can the impact factor measure the quality of research?

**DOI:** 10.3402/qhw.v7i0.19772

**Published:** 2012-10-11

**Authors:** Lillemor Hallberg

*International Journal of Qualitative Studies on Health and Well-being (QHW*) strives hard to maintain high scientific quality. As soon as a manuscript is submitted to the journal, the Chief Editor reads it to see: (i) whether it is within the scope of the journal and (ii) if it is scientifically and methodologically of such quality that it is worth asking at least two peers for a rigorous review. If the answer to both questions is “yes”, then the Co-editor who has most experience in the particular research area of the study will take care of the continued review process.

Beyond the Chief Editor, *QHW* at present has three Co-editors with extensive knowledge in the field of health and well-being and long research experience. In addition, the journal has an impressive international Editorial board as well as a long and ever-increasing list of peer-reviewers. These persons are all renowned researchers in different research areas as well as experts of different qualitative research approaches. At least two, most often three, independent reviewers are requested to review each manuscript. The review process is “double blind”—that is, the author and the reviewers are unknown to each other—and we are constantly striving to make the process as short and smooth as possible. Based on a balanced evaluation of the reviewers’ comments, the editorial decision will be either: (i) acceptance; (ii) acceptance after revision; (iii) revision and resubmission; (iv) rejection; or (v) submission elsewhere. At present, the rejection rate is close to 50%.

*QHW* is accepted for indexing with the Science Citation Index through Web of Science and has also received its first official Impact Factor (for 2011). Also, recently the journal was accepted for indexing in MEDLINE. This, along with indexing with PubMed, Scopus, and PsychInfo, among others, and archiving in PubMed Central, CLOCKSS, and Portico are obvious signs of the journal's generally recognized high quality.

An Impact Factor is based on citations to articles in the journal during the previous 2 years. In this way, *QHW's* Impact Factor for 2011 of 0.481 is based on citations to articles published in the journal during 2009 and 2010. By calculating and dividing two measures, which can be named A and B, the Impact Factor is thus paradoxically a quantitative way of expressing a journal's quality:

- Measure A is the number of times that articles published in a certain journal during a certain period (in this case 2009 and 2010) are cited during the following year (in this case 2011).
- Measure B is the total number of citable articles (for instance, Editorials and Letters to Editor are not included) in the journal during the same period, that is, 2009 and 2010.
- The Impact Factor is calculated by dividing the sum of measure A with the sum of measure B.

But is this also a qualitative measure? Can we automatically assume that articles published in a journal with a high Impact Factor are of higher scientific quality than articles published in a journal with a lower Impact Factor? The simple answer is no: the Impact factor is not a reliable instrument for measuring quality of research. In the first place, I believe that—for many reasons—there is a risk that papers of low or modest quality may be more frequently cited than, as would perhaps be expected, papers of high quality. Also, a journal's high Impact Factor may be based on citations to just a few of the articles published therein, leaving the remainder with few or no citations. In addition, editorial policy can affect a journal's Impact Factor. Review articles, methodological articles, and studies on “hot” topics can be selected for publication because it is a well-known fact that these articles are cited more often than empirical studies. Such a policy is of course likely to result in an increasing Impact Factor for the journal as such. But what does it say about the quality of the published articles?

*QHWs* 2011 Impact Factor of 0.481 looks quite modest in comparison to that of many medical journals. In fairness it should be said that it is based on citations during a period that was was partly characterized by a restricted number of readers. In 2009, the journal was only available to relatively few subscribers, most often libraries at universities. At this time, many universities due to financial restraints discontinued subscriptions and, unfortunately, journals publishing interdisciplinary qualitative research on health and well-being were most often not prioritized for continued subscription. As from 2010, *QHW* has been published under an Open Access model with no restriction to access: Readers all over the world now have the possibility to access and download published articles in full text, free of charge. The shift to Open Access has proved a great success for *QHW* with a considerable increase in readers which, in turn, makes the journal an attractive publication venue for publishing authors. During 2011, the journal had 25,846 visits to the website from readers in 142 countries; the number of downloads of full text articles from the entire archive were more than 100,000 (including through PubMed Central). We believe that free access to all content will eventually lead to a considerably increased Impact Factor over the coming years. However, we also constantly work to improve *QHW's* quality.

Actually, what does quality of qualitative research include? How can quality of research be judged or measured, for instance when peers undertake to review manuscripts? Are the quality criteria the same for all scientific branches—quantitative as well as qualitative studies? Can we talk about generalizability, validity, reliability and replication when it comes to qualitative studies?

My opinion is that it is indeed possible to replicate a qualitative study. If two different researchers have the same research question, the same theoretical perspective, and the same principles of selecting informants and collecting and analyzing data, there is reason to believe that the results of the studies will be the same—or at least very similar. Objectivity in a study depends on the researcher′s ability to avoid being governed by preconceptions and/or wishful thinking when analyzing and interpreting data. Of course it is essential to differentiate between the emerging results and one′s own values and wishes. Transferability of results from a qualitative study to groups other than the ones under study will increase if systematic and broad sampling has been done as this secures that variation in data has been captured. In addition, transferability should also be grounded in and supported by theoretical reasoning.

Calculation of an Impact Factor is a complicated process but hardly considers any of these important quality aspects. We continually work to publish articles that meet the criteria for high scientific quality. If, eventually, this leads to a higher Impact Factor, we have nothing against that.

*Lillemor Hallberg*, Professor emerita Chief Editor

